# Successful treatment of advanced squamous cell carcinoma arising from mature cystic teratoma of the ovary with homologous recombination deficiency: A case report

**DOI:** 10.1016/j.ijscr.2023.108329

**Published:** 2023-05-20

**Authors:** Ryo Tamura, Masayuki Yamaguchi, Yohei Kitani, Nobumichi Nishikawa, Takashi Kawasaki, Akira Kikuchi

**Affiliations:** a Department of Gynecology, Niigata Cancer Center Hospital, Niigata, Japan; b Department of Obstetrics and Gynecology, Niigata University Graduate School of Medical and Dental Sciences, Japan; c Department of Pathology, Niigata Cancer Center Hospital, Niigata, Japan

**Keywords:** Mature cystic teratoma, Squamous cell carcinoma, Olaparib, Bevacizumab, Homologous recombination deficiency, Platinum-based chemotherapy

## Abstract

**Introduction and importance:**

Squamous cell carcinoma (SCC) arising from mature cystic teratoma of the ovary (MCT-SCC) has a poor prognosis at advanced stages. Although the relationship between homologous recombination deficiency (HRD) and platinum-based chemotherapy sensitivity or poly (ADP ribose) polymerase (PARP) inhibitor efficacy in epithelial ovarian cancer has been demonstrated in clinical trials, the significance of HRD status in MCT-SCC has not previously been described.

**Case presentation:**

A 73-year-old woman underwent emergency laparotomy due to ovarian tumor rupture. The ovarian tumor was strongly adherent to the surrounding pelvic organs and could not be completely resected. The postoperative diagnosis was stage IIIB MCT-SCC (pT3bNXM0) of the left ovary. After surgery, we conducted the myChoice CDx. The genomic instability (GI) score of 87 was remarkably high, and there was no *BRCA1*/*2* pathogenic mutation. After six courses of combination therapy with paclitaxel and carboplatin, the residual tumors had shrunk by 73 %. We performed interval debulking surgery (IDS), and the residual tumors were completely resected. Subsequently, the patient underwent two courses of the combination of paclitaxel, carboplatin, and bevacizumab, followed by maintenance therapy with olaparib and bevacizumab. Twelve months after IDS, no recurrence has been observed.

**Clinical discussion:**

The present case suggests that there are some HRD cases among MCT-SCC patients and that IDS and maintenance therapy with PARP inhibitors may be effective in such cases, as in epithelial ovarian cancer.

**Conclusion:**

Although the frequency of HRD-positive status in MCT-SCC remains unknown, HRD testing may provide appropriate treatment options for advanced MCT-SCC.

## Highlights

• Squamous cell carcinoma arising from mature cystic teratoma of the ovary (MCT-SCC) has a poor prognosis at advanced stages.
• The significance of the homologous recombination deficiency status in MCT-SCC remains unclear.
• We presented the case of an MCT-SCC patient with a remarkably high genomic instability score who was successfully treated.

## 1 Background

The malignant transformation of mature cystic teratoma of the ovary is a rare condition, and about 80 % of cases were squamous cell carcinoma (SCC). Although complete cytoreductive surgery, including hysterectomy, bilateral salpingo-oophorectomy, and lymphadenectomy followed by adjuvant platinum-based chemotherapy, is the standard of care for SCC arising from mature cystic teratoma of the ovary (MCT-SCC), stage II and higher cases of MCT-SCC have poor prognoses with five-year overall survival < 50 % [1,2].

Approximately 50 % of cases of ovarian cancer harbor a homologous recombination deficiency (HRD) due to alterations of homologous recombination repair pathway genes [3]. The relationship between HRD and platinum-based chemotherapy sensitivity or poly (ADP ribose) polymerase (PARP) inhibitor efficacy in ovarian cancer has been demonstrated in previous clinical trials [4,5]. The National Comprehensive Cancer Network (NCCN) guidelines recommend implementing BRCA and HRD testing in patients with advanced ovarian cancer [6]. However, the significance of HRD status or homologous recombination repair pathway gene abnormalities in rare forms of ovarian cancer such as MCT-SCC is unclear.

Here, we present the case of a patient with advanced MCT-SCC and a remarkably high genomic instability (GI) score. The patient received maintenance therapy with olaparib and bevacizumab after platinum-based chemotherapy and two sessions of debulking surgery.

## 2 Case presentation

**Fig. 1:**
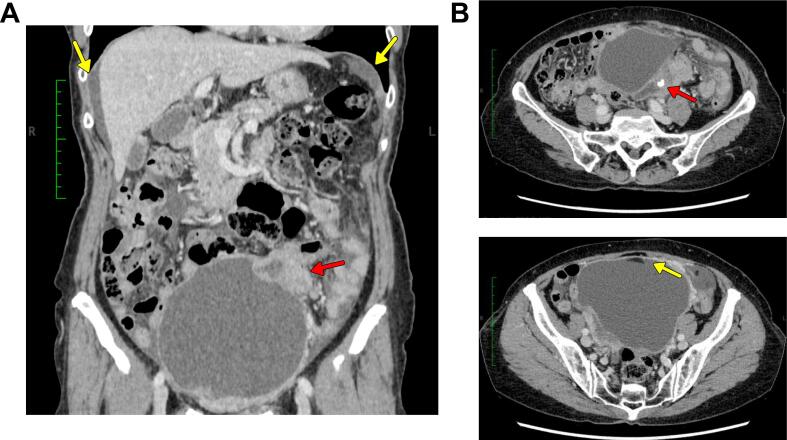
Computed tomography (CT) imaging before surgery. **A** Coronal CT image. A 10-cm-sized pelvic tumor with a solid part is detected (red arrow). Ascites extending to the upper abdomen is also detected (yellow allow). **B** Axial CT images. The tumor lacks turgidity and is suspected to have ruptured. Fatty components and calcifications are detected within the tumor. (For interpretation of the references to colour in this figure legend, the reader is referred to the web version of this article.)

**Fig. 2:**
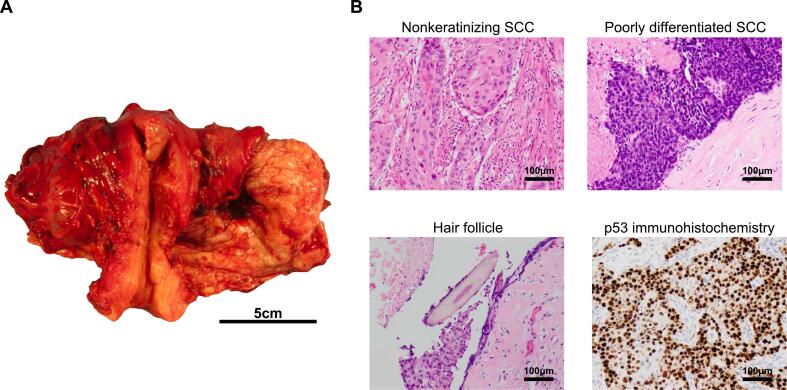
Histological and immunohistochemical findings. **A** Macroscopic findings of the primary tumor. The left ovarian tumor has a solid part (red arrow). **B** Representative images of H&E staining and p53 immunohistochemical staining are shown (200 × magnifications). Most of the tumor is nonkeratinizing squamous cell carcinoma (SCC) and partially low differentiated SCC. Scattered hair follicles are present (red arrow). p53 overexpression in the nonkeratinizing SCC part is confirmed. (For interpretation of the references to colour in this figure legend, the reader is referred to the web version of this article.)

**Fig. 3:**
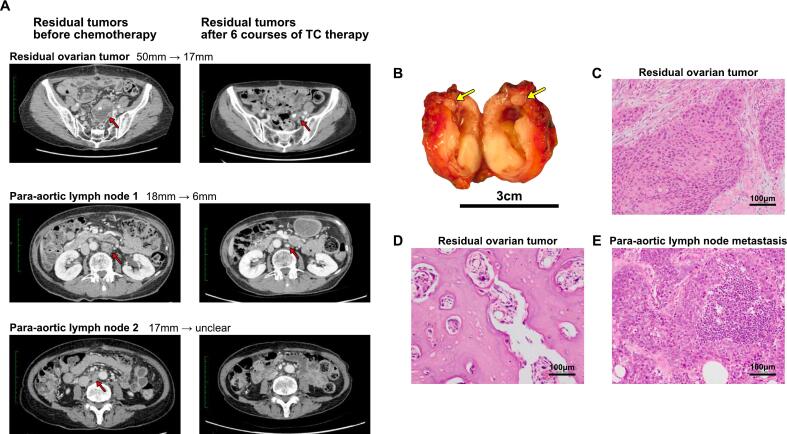
Computed tomography (CT) imaging and histological findings of residual tumors. **C**T images taken before and after chemotherapy. Red arrows show the change of target lesions (residual ovarian tumor, para-aortic lymoh nodes). **B** Macroscopic findings of the residual tumor. The yellow arrow indicates the ureter entrapped by the tumor. **C, D** Representative images of H&E staining of the residual tumor. Nonkeratinizing squamous cell carcinoma (SCC) is detected alongside the primary ovarian tumor (**C**). The chemotherapy-induced hyalinized stroma is widely observed (**D**). **E** Metastatic SCC is detected in a para-aortic lymph node. (For interpretation of the references to colour in this figure legend, the reader is referred to the web version of this article.)

A 73-year-old multiparous woman with no remarkable past medical history visited her local physician with symptoms of low abdominal pain. A left ovarian tumor measuring approximately 15 cm was identified, and she was referred to our hospital for further examination and treatment. Computed tomography (CT) imaging revealed a complex left ovarian tumor comprised of solid and fat components and calcifications with para-aortic and pelvic adenopathy. The blood squamous cell carcinoma antigen (SCC-Ag) level of 11.6 ng/ml was high. Based on these findings, she was suspected of having MCT-SCC. One week after the initial visit, she presented with increasing abdominal pain. Blood tests revealed elevated levels of inflammatory markers (white blood cell [WBC]): 13,100/μl, C-reactive protein (CRP) (16.83 mg/1), and CT imaging revealed that the tumor had shrunk to 10 cm in size with ascites extending into the upper abdomen (Fig. 1). The decision was made to proceed an emergency exploratory laparotomy which included total abdominal hysterectomy, bilateral salpingo-oophorectomy, and partial omentectomy. The left ovarian tumor measured 10 cm and had ruptured, and the ensuing ascitic fluid cytology was positive. Intraoperatively, there were multiple unresectable 5 mm peritoneal implants, significant adhesions, and extensive adenopathy resulting in suboptimal debulking. The patient was in poor general condition due to tumor rupture, and maximal effort, including bowel resection, was impossible due to emergency surgery. Postoperatively, pathology revealed poorly differentiated nonkeratinizing SCC consistent with MCT-SCC having p53 overexpression with omental metastasis, conferring stage IIIB ovarian cancer (pT3bNXM0) (Fig. 2). Interestingly, there was a synchronous stage IA grade 1 endometrioid adenocarcinoma of the uterus. After surgery, the myChoice CDx (Myriad Genetic Laboratories, Inc., Salt Lake City, United States) test and microsatellite instability (MSI) test (SRL, Tokyo, Japan) were performed using specimens from the nonkeratinizing SCC section of the primary tumor. The GI score of 87 was remarkably high, and there was no *BRCA1*/*2* pathogenic mutation. The MSI test was negative. Three weeks after surgery, the patient began intravenous (IV, AUC6) carboplatin and paclitaxel (IV, 175 mg/m^2^). CT imaging after six courses showed that residual tumors had shrunk by 73 % (target lesions: 85 mm to 23 mm; Fig. 3A) and blood SCC-Ag levels decreased from 12.8 ng/ml postoperatively to 1.6 ng/ml after one course of chemotherapy, and remained in the normal range thereafter. Since complete resection of the residual tumor was considered feasible, and maximal effort was not possible at the initial surgery, we decided to perform interval debulking surgery (IDS). We performed IDS six months after the first visit. R0 resection was achieved with pelvic and paraortic lymphadenectomy and partial ureteral resection. We did not perform hyperthermic intraperitoneal chemotherapy. The pathology was consistent with metastatic SCC (Fig. 3B–E). Since six weeks after IDS, the patient received two additional courses of paclitaxel, carboplatin, and bevacizumab (IV, 15 mg/m^2^) and was given bevacizumab and olaparib (600 mg/day). Twelve months after IDS, no recurrence has been observed.

## 3 Discussion

Although platinum-based chemotherapy shows some efficacy for MCT-SCC, the prognosis of advanced cases remains poor [1,2]. Clinical biomarkers that predict the efficacy of platinum-based chemotherapy for MCT-SCC have not been identified. Here, we present the case of an MCT-SCC patient with a remarkably high GI score who was successfully treated with two debulking surgeries, platinum-based therapy, and maintenance therapy with olaparib and bevacizumab. MCT-SCC is often refractory to chemotherapy, and the therapeutic significance of IDS for MCT-DCC is unclear. In the present case, we considered complete removal of the residual tumors, which would be feasible after six cycles of platinum-based chemotherapy; we decided to perform IDS based on the treatment protocol of epithelial ovarian cancer after explaining the option of continuing chemotherapy to the patient. Homologous recombination deficiency is a biomarker for response to platinum-based chemotherapy and PARP inhibitors not only in ovarian cancer, but also in breast, prostate, and pancreatic cancer [7].Moreover, HRD in SCC is rare but has been described, and the efficacy of PARP therapy has been tested in preclinical and clinical models for lung SCC [8]. Additionally, bevacizumab maintenance therapy may be effective in MCT-SCC [9]. The combination of bevacizumab and olaparib demonstrated efficacy in a phase III trial for HRD-positive epithelial ovarian cancer [4]. This case suggests that HRD status may be critical in determining the treatment strategy for MCT-SCC as well as other cancers.

In MCT-SCC, the frequency and mechanisms of HRD were unknown. MCT-SCC with *BRCA1*/*2* pathogenic mutations has not been reported yet. The cause of the high GI score may be related to the molecular biological features of MCT-SCC. Recently, two comprehensive analyses have shown a characteristically high frequency of tumor suppressor protein P53 (*TP53)* pathogenic mutations in MCT-SCC, 20/25 (80 %) and 7/8 (87.5 %), respectively [10,11]. In this case, the p53 mutation was suggested by immunostaining. *TP53* mutations promote two types of genomic instability, which are chromosomal and amplification instability [12]. Recently, an association between *TP53* mutations and HRD status has been reported [13]. In MCT-SCC, *TP53* wild-type patients have a poorer prognosis than *TP53*-mutant patients [10], which may also be related to HRD status and chemotherapy sensitivity.

In the present case, the MSI test was negative; however, a mismatch repair deficiency was demonstrated in two of seven carcinomas arising from ovarian teratoma [14]. Furthermore, more than half of MCT-SCC cases demonstrated programmed death receptor-1 (PD-1) expression and high tumor-infiltrating CD8-positive lymphocyte counts, suggesting that immune checkpoint inhibitors may be effective for MCT-SCC [11]. In a recent study, HRD is inversely correlated with MSI and is frequently identified in immunologically cold tumors [15]. Therefore, a PARP inhibitor may be an attractive therapeutic option in patients who do not respond to the anti-PD-1 antibody.

In this case, there was a synchronous endometrioid adenocarcinoma of the uterus. Although genetic testing of the uterine tumor was not performed, the synchronous occurrence of different tumors suggests that a specific germline gene mutation may have occurred in the patient. Further clarification of the association between HRD status and molecular biological characteristics of MCT-SCC may provide more appropriate treatment options for MCT-SCC.

To the best of our knowledge, this is the first reported case of MCT-SCC with HRD-positive status. Although the frequency of HRD in MCT-SCC remains unknown, HRD testing may provide appropriate treatment options for some cases of advanced MCT-SCC.

## Consent

Written informed consent was obtained from the patient for publication of this case report. A copy of the written consent is available for review by the Editor-in-Chief of this journal on request.

## Ethical approval

This study was performed under the Declaration of Helsinki and was approved by the institutional ethics review board at Niigata Cancer Center (No 1148) on October 1, 2021.

## Funding

This work was supported in part by JSPS KAKENHI grant number 21K16785 (Grant-in-Aid for Young Scientists for Ryo Tamura), the Takeda Grant for Ryo Tamura and the Kanzawa Grant for Ryo Tamura.

## Guarantor

Ryo Tamura.

## Research registration number

Not applicable.

## CRediT authorship contribution statement

**Ryo Tamura:** Conceptualization, Methodology, Formal analysis, Investigation, Writing – original draft. **Masayuki Yamaguchi:** Writing – review & editing. **Yohei Kitani:** Writing – review & editing. **Nobumichi Nishikawa:** Writing – review & editing. **Takashi Kawasaki:** Writing – review & editing. **Akira Kikuchi:** Writing – review & editing, Supervision.

## Declaration of competing interest

The authors declare that they have no known competing financial interests or personal relationships that could have appeared to influence the work reported in this paper.
